# Transarterial Chemoembolization and Unresectable Hepatocellular Carcinoma: A Narrative Review

**DOI:** 10.7759/cureus.28439

**Published:** 2022-08-26

**Authors:** Nisha Manjunatha, Vinutna Ganduri, Kruthiga Rajasekaran, Shrimahitha Duraiyarasan, Mayowa Adefuye

**Affiliations:** 1 Research, Our Lady of Fatima University College of Medicine, Metro Manila, PHL; 2 Research, Bhaskar Medical College, Hyderabad, IND; 3 Research, Rajah Muthiah Medical College and Hospital, Chidambaram, IND; 4 Research, K.A.P. Viswanatham Government Medical College, Tiruchirappalli, IND; 5 Research, University of Ibadan College of Medicine, Ibadan, NGA

**Keywords:** child-pugh (c-p) classification, transcatheter arterial embolization, radio-frequency ablation, deb-tace, tace complication, neoadjuvant chemoembolization, barcelona clinic liver cancer stage, unresectable hcc, transarterial chemoembolization (tace), hepatocellular carcinoma (hcc)

## Abstract

Hepatocellular carcinoma (HCC) is an aggressive tumor, and even with the breakthrough in preventive strategies, and new diagnostic and treatment modalities, incidence and fatality rates continue to climb. Patients with HCC are most commonly diagnosed in the later stage, where the disease has already advanced, making it impossible to undertake potentially curative surgery. Transarterial chemoembolization (TACE) is a locoregional therapy regarded as a first-line treatment in patients with intermediate-stage HCC (Barcelona clinical liver cancer {BCLC}-B). TACE is a minimally invasive and non-surgical procedure that combines local chemotherapeutic drug administration with embolization to treat HCC. It helps limit tumor growth, preserve liver function, and increase overall and progression-free survival in patients with intermediate-stage HCC. This article has reviewed the efficacy, survival, limitations, and overall benefit of TACE in patients with unresectable HCC. This article has also discussed the effectiveness of TACE for neoadjuvant chemoembolization and the use of TACE with combination therapies.

## Introduction and background

Hepatocellular carcinoma (HCC) is the most prevalent hepatobiliary malignancy arising from liver parenchymal cells in the context of liver cirrhosis which develops as a result of chronic liver injury, leading to subsequent hepatocyte regeneration, aberrant nodules development, and fibrosis formation [1,2]. In 2020, HCC accounted for 8.3% of deaths, making it the third leading cause of cancer-related mortality worldwide [3]. In terms of global incidence, HCC is the fifth most common cancer, with an estimated 905,677 individuals affected each year globally. However, many infected people go undiagnosed as chronic infections are generally asymptomatic [2,3]. With men being afflicted twice as often as women, HCC has the highest incidence rates in transitioning countries such as South-East Asia, East Asia, and Sub-Saharan Africa; Mongolia has the most increased occurrence and mortality rate [3]. The significant risk factors for HCC are hepatitis B virus (HBV), hepatitis C virus (HCV), diabetes mellitus type 2, obesity, non-alcoholic fatty liver disease (NAFLD), α1-antitrypsin deficiency, hemochromatosis, autoimmune diseases, environmental exposures like aflatoxin-contaminated food, tobacco, and heavy alcohol consumption [4]. HBV and HCV continue to be the most significant etiological agents in developing chronic hepatitis [5]. Inflammation and oxidative DNA damage in liver injury in chronic liver disease induce critical genetic mutations that may also lead to malignant transformation [1]. HCC commonly presents with an enlarged liver, right upper quadrant pain, weight loss, sudden hepatic decompensation, and worsening hepatic function secondary to cirrhosis and portal vein invasion in large tumors [6]. American Association for the Study of Liver Disease (AASLD) and the European Association for the Study of the Liver (EASL) recommend that early monitoring in high-risk patients such as those with chronic HBV and cirrhosis related to any etiology are associated with an increased survival rate [7,8]. Although the preferred modality for HCC surveillance is abdominal ultrasound (US), computed tomography (CT) and magnetic resonance imaging (MRI) are superior in terms of sensitivity and specificity [7-9]. The biomarker alfa fetoprotein (AFP) has poor sensitivity and specificity due to a false positive rate, but when used in conjunction with the US, it had significantly higher sensitivity in the detection of early-stage HCC than the US alone, the other biomarker that is also used in the detection of HCC is des-gamma-carboxy-prothrombin (DCP) [10]. Hepatic resection (HR) in patients with HCC depends on the tumor size, location, and liver function, thereby making it the first-line treatment for non-cirrhotic patients with solitary tumors confined to the liver with no vascular invasion and good liver function [4]. Liver transplantation (LT) is optimal in patients who fall under the Milan Criteria with a single HCC <5 cm or two to three tumors each of <3 cm, removing both malignant and pre-malignant lesions [2]. HCC is frequently diagnosed when the disease has progressed to an advanced stage where no effective treatment would improve survival [4]. Locoregional therapies such as ablation, which includes percutaneous ethanol injection (PEI), radiofrequency ablation (RFA), and intra-arterial therapy such as transarterial chemoembolization (TACE), are treatment modalities used when the patient is not an appropriate candidate for HR or LT [2]. TACE is the preferred locoregional treatment of choice in patients with large unresectable tumors and multiple lesions within the liver. It is used in neoadjuvant chemoembolization, which helps down-size large tumors to fit into HR or LT criteria [11,12]. The liver has a dual blood supply, whereas the hepatic artery becomes the predominant blood supply to the tumor as it grows in size [2,4]. TACE is performed by injecting a chemotherapeutic agent (doxorubicin or cisplatin) under radiological guidance, followed by embolizing the hepatic artery supplying the tumor with lipiodol or gel foam resulting in selective hypoxia and necrosis in cancer; it also minimizes systemic exposure while maximizing hepatic concentrations of chemotherapeutic agents [2,4]. In individuals with cirrhotic HCC with vascular invasion, TACE has been demonstrated to increase overall survival and decrease the recurrence rate of HCC [12]. This review aims to provide a comprehensive overview of published studies on TACE in the treatment of unresectable HCC and build an evidence-based practice for this palliative treatment in the management of HCC.

## Review

Evolution of TACE

The primary notion of TACE relies upon the concept of the liver's dual blood supply; a bulk of blood flow to the hepatic tumors comes from the hepatic artery; therefore, a significant proportion of drugs reaching the tumor region by intra-arterial application of embolizing agent block the blood supply and amplify the cytotoxic impact on tumor cells and reduces adverse effects of chemotherapy [13,14]. According to Park et al., HCC tumorigenesis was a process that included parenchymal arterialization, growth of unpaired arteries, which was an essential part of neogenesis, and sinusoidal capillarization leading to gradually transitioning the blood supply from portal to arterial [13,15]. Mori et al. in 1966 were the first to perform hepatic artery ligation to create ischemic necrosis of the tumor leading to regression. Still, due to the rapid formation of collateral circulation, the impact of this procedure was relatively short [13]. Tumor embolization, one of the techniques of therapeutic embolization that involves obstructing blood supply to the tumors by using embolic agents injected via a percutaneous injection, was used by angiographers in the early 1970s to treat hepatic tumors [14]. Later in 1974, Doyon et al. were the first to perform and describe TACE [16]. Intra-arterial injections of adriamycin, 5-fluorouracil, and mitomycin-c in combination or alone were utilized in the late 1970s; one dose of these drugs intra-arterially was highly superior to multiple high doses through the systemic route [17-19].

Today, TACE uses two techniques - conventional TACE (cTACE) and TACE with drug-eluting beads (DEB-TACE) [20]. Intra-arterial injection of cytotoxic agents such as doxorubicin, cisplatin, or lipiodol is utilized in cTACE, followed by embolic agents like gelatin sponge. The drugs cause embolization of the microcirculation of the tumor [20]. Based on using non-absorbable microspheres that use ionic bonds to store and gradually release drugs, DEB-TACE ensured greater distal embolization of tiny vessels with the usage of particles, resulting in a highly selective extraction and persistent closure of tumor feeding vessels [20-22]. The most commonly used cytotoxic drugs in TACE are doxorubicin, cisplatin, and epirubicin as single agents combined with lipiodol that carry these drugs to the tumor [23]. An interventional radiologist or a gastroenterologist usually performs TACE through a percutaneous transarterial approach where the femoral artery on the right is punctured, and under local anesthesia, an arterial sheath is placed into the artery by Selinger's method; a catheter is then introduced through the sheath; a wire often aids the catheter into the arterial system under imagining guidelines [14]. To reach the target branches that supply the tumor, the catheter is then inserted through the common hepatic artery and then the proper hepatic artery; after locating the feeding vessel, an iodized oil along with cytotoxic drug, followed by embolization particles is injected [14].

TACE and the Barcelona clinical liver cancer staging

Patient selection is one of the most crucial factors in TACE [24]. The Barcelona clinical liver cancer (BCLC) staging has been universally acknowledged in medical practice and is also employed in many clinical trials; it is far superior in predicting prognosis than other staging systems (Table 1) [24-26]. EASL and AASLD also prefer BCLC for HCC staging as it plays an essential role in determining the overall survival and planning potential treatment options according to the patient's profile; it also plays a role in strengthening prognostic and stage-specific therapeutic management [8,25]. BCLC provides management options for very early, early, intermediate, advanced, and terminal tumors (Table 1) [26]. However, in a clinical setting, along with the BCLC staging, the patient's status is evaluated by an inter-disciplinary tumor panel and the physician in charge who will also consider other factors such as cancer burden and extension, age, chronic conditions, socioeconomic status, and sentient values and principles [24]. The BCLC staging for HCC is mentioned in Table 1.

**Table 1:** BCLC staging, management options, and survival. BCLC: Barcelona clinical liver cancer; BSC: best supportive care; HCC: hepatocellular carcinoma; PS: performance status; TACE: transarterial chemoembolization

Hepatocellular carcinoma				
Very early stage (0)	Early stage (A)	Intermediate stage (B)	Advanced stage (C)	Terminal stage (D)
Single <2 cm	Single or <3 nodules each <3 cm	Multinodular	Portal invasion and/or extrahepatic spread	Any tumor burden
Child-Pugh A	Child-Pugh A-B	Child-Pugh A-B	Child-Pugh A-B	Child-Pugh B-C
PS 0	PS 0	PS 0	PS 1-2	PS 3-4
Ablation/resection/transplantation		TACE	Systemic therapy	BSC
Survival >5 years		Survival >2.5 years	Survival 2 years	Survival 3 months

The BCLC system utilizes elements like the size and number of tumors in the liver, a general physical status called the performance status by the Eastern Co-operative Oncology Group (ECOG), which also aids in strongly predicting long-term survival in patients with both cirrhosis and tumors (Table 2) [27,28]. The performance status by ECOG is mentioned in Table 2.

**Table 2:** Performance status assessed by the ECOG ECOG: Eastern Cooperative Oncology Group

Grade	ECOG performance status
0	Fully active, no restriction in the performance
1	Physical strenuous activity restricted; completely ambulatory and able to carry out light work of sedentary nature
2	Capable of self-care but unable to carry out any work activities; >50% of waking hours, ambulatory
3	Capable of limited self-care only; confined to bed or chair >50% of waking hours
4	Completely disabled; cannot carry out any self-care; wholly confined to bed or chair
5	Dead

The liver's functional status is assessed by using the Child-Pugh score which also predicts post-operative outcome in patients with HCC (Table 3) [29,30]. Patients with early-stage cancer are often candidates for HR, RFA, and LT; TACE is considered an alternative for these treatment modalities when the patient cannot benefit from them due to a lack of live donors, poor liver function, and damage to the normal liver tissues during resection [26]. Child-Pugh criteria are mentioned in Table 3.

**Table 3:** Child-Pugh criteria are used to assess hepatic liver function, the severity of cirrhosis, and a predictor for post-operative mortality. Child-Pugh A: 5-6 points, well-compensated liver; Child-Pugh B: 7-9 points, moderately impaired hepatic function; Child-Pugh C: 10-15 points, decompensated liver. INR: international normalized ratio

	1 point	2 points	3 points
Encephalopathy	None	Grade 1 and 2	Grade 3 and 4
Ascites	None	Slight	Moderate
Bilirubin	<2 mg/ml	2-3 mg/ml	>3 mg/ml
Albumin	>3.5 mg/ml	2.8-3.5 mg/ml	<2.5 mg/ml
Prothrombin time/INR	<4 s/<1.7	4-6 s/1.7-2.2	>6 s/>2.2

A retrospective study conducted for nine years with a sample population of 2247 patients with HCC, BCLC stage A or B of varying sizes showed that there was overall survival and progression-free survival in patients at one, three, five, and eight years when TACE was combined with RFA than TACE alone, thereby concluding that the combination was effective regardless of the size (Table 4) [31]. TACE is the recommended treatment approach for intermediate stage (BCLC-B) HCC, which comprises large asymptomatic or numerous tumors with no invasion of vascular channels or extrahepatic metastasis; the intermediate stage is further classified based on tumor burden (Table 1) [24,26]. The first group under BCLC-B is included under the extended LT criteria, the criteria include patients with well-defined tumors; the second group is patients with specified tumor burden and a good portal flow, therefore, being good candidates for TACE [24]. The BCLC third subgroup consists of patients with extensive HCC, which is comprehensive and widespread; systemic therapy is the recommended management option [24]. Varghese et al. conducted an observational study for four years with a sample population of 124 patients with HCC in BCLC stages B/C. It was found that TACE, along with sorafenib, was more efficacious than sorafenib or TACE alone by reducing the progression of tumor from 83.3% to 37.8%, improved partial response (43.2% vs. 3.3%), and showing significant overall survival from nine months to 16 months (Table 4) [32]. In patients with HCC and portal vein tumor thrombosis (PVTT), TACE, combined with radiotherapy, has shown improved results by conserving portal blood flow, thereby delaying the deterioration of liver function and intra-vascular tumor growth [26]. Patients with terminal stage HCC with most tumor-related symptoms and impaired liver function are not candidates for LT; best supportive care (BSC) is the preferred management option as other treatments would not increase the patient's survival [24]. Xiang et al. conducted a retrospective study on a sample population of 1040 patients with HCC with PVTT who were classified with Cheng's PVTT classification. The study showed that patients with PVTT types 1-3 were associated with a better overall survival when treated with TACE than with BSC; regardless of whether BSC or TACE was employed, PVTT type 4 showed the worst outcome (Table 4) [33]. A summary of all the studies related to the utilization of TACE in patients with HCC is listed in Table 4.

**Table 4:** TACE with non-TACE combination therapy compared to TACE alone/non-TACE modalities in the management of HCC. BCLC: Barcelona clinical liver cancer; BSC: best supportive care; PVTT: portal vein tumor thrombosis; RFA: radiofrequency ablation; TACE: transarterial chemoembolization; HCC: hepatocellular carcinoma

References	Design	Subjects	Criteria for inclusion	TACE alone/TACE with non-TACE combination therapy	TACE alone/non-TACE	Outcome
Ren et al. (2019) [31]	Retrospective study	2447	BCLC stages A and B	TACE with RFA		Increased overall survival and progression-free survival in patients at one, three, five, and eight years when TACE was combined with RFA than TACE alone, concluding that the combination was effective regardless of the size.
Varghese et al. (2017) [32]	Observational study	124	BCLC B and C	TACE with sorafenib	TACE alone and sorafenib alone	TACE, along with sorafenib, was more efficacious than sorafenib or TACE alone by reducing the progression of tumor from 83.3% to 37.8%, improved partial response (43.2% vs. 3.3%), and showed significant overall survival from 9 months to 16 months.
Xiang et al. (2019) [33]	Retrospective study	1040	HCC patients with PVTT	TACE	BSC	Patients with PVTT type 1-3 were associated with a better overall survival when treated with TACE than BSC, regardless of whether BSC or TACE was employed, PVTT-4 showed the worst outcome.

TACE and unresectable HCC

TACE is the first-line therapy for unresectable, intermediate, and advanced-stage HCC patients [34]. Patients with a maintained liver function, with multinodular or isolated large tumor >3 cm without extrahepatic metastasis, vascular invasion, or cancer-related symptoms who are ineligible for percutaneous or surgical management are said to be the best candidates for TACE (Table 3) [35]. TACE, in combination with RFA, microwave ablation (MWA), and cryoablation (CRA), is far more effective than TACE alone [34]. 

A retrospective study was conducted for seven years on a sample population of 108 patients with unresectable HCC, further divided into the TACE-MWA group and the TACE-CRA group; the study found that the median survival of both the groups (20.9 months vs. 13 months) with unresectable HCC was increased. TACE combined with MWA also decreased the rate of complication occurrence (66% vs. 88.3%) (Table 5) [34]. In a study conducted by Yoon et al. for three years with a sample population of 90 patients with HCC who had macrovascular invasion found that when compared to a group that uses sorafenib alone, TACE with radiotherapy (RT) offered better progression-free survival (87% vs. 34.3%) and was very well tolerated with a higher radiological response rate (33.3% vs. 2.2%) and overall survival (55 weeks vs. 43 weeks) (Table 5) [36]. Therefore when compared to TACE alone, a combination of TACE with local treatments showed a much better outcome in the survival of patients with unresectable HCC [37]. Doxorubicin is one of the most efficient chemotherapy agents used in HCC; it works by intercalating with topoisomerase II, resulting in alterations of chromatin structure and the production of free radicals leading to oxidative damage [38]. A retrospective study with a sample population of 54 patients with unresectable HCC with TACE combined with doxorubicin eluting microspheres (DEB-TACE) showed that the drug is safe and effective with better median survival at six months, one year, and two years at a percentage of 77%, 59%, and 32%, respectively, and overall median survival of 95%; DEB-TACE was well tolerated in patients with unresectable HCC (Table 5) [39]. Vascular endothelial growth factors (VEGF) play a crucial role in developing HCC, and their expression has been linked with tumor size and grade [40]. Sorafenib is a multikinase inhibitor that primarily acts on vascular endothelial growth factor receptors (VEGFR 2-3) [40]. TACE induces hypoxia and ischemic necrosis to the local tissues, activating hypoxia-inducible factors that raise VEGF, an endothelial cell mitogen, causing neovascularization and tumor recurrence. As a result, supplementing TACE with anti-angiogenic drugs showed to be a helpful method of improving outcomes [8]. Kudo et al. conducted a randomized, multicentre, prospective trial with a sample population of 156 patients with unresectable HCC. One group was subjected to TACE alone, and the other group with TACE combined with sorafenib. The study found that patients with TACE combined with sorafenib had a significant median progression-free survival (25.2 months vs. 13.5 months), the median time to untreatable progression (TTUP) ( 26.7 months vs. 20.6 months), and better overall survival at one and two years (96.2% and 82.7%) than TACE alone (77.2% and 64.6%) (Table 5) [41]. Providing sorafenib following TACE stems from the finding that HCC recurrence after TACE may be caused by an increase in VEGF production, resulting in new vasculature formation in the residual tumor [40]. A summary of all the studies related to the utilization of TACE in the management of unresectable HCC is mentioned in Table 5.

**Table 5:** TACE with non-TACE combination therapy vs. TACE alone/TACE with non-TACE combination therapy/non-TACE in managing unresectable HCC. BCLC: Barcelona clinical liver cancer; BSC: best supportive care; DEB-TACE: drug-eluting beads transarterial chemoembolization; HCC: hepatocellular carcinoma; MWA: microwave ablation; RT: radiotherapy; TACE: transarterial chemoembolization; RFA: radiofrequency ablation; TTUP: time to untreatable progression

References	Design	Subjects	Criteria for inclusion	TACE alone/TACE with non-TACE/non-TACE	TACE alone/TACE with non-TACE/ non-TACE	Outcome
Wei et al. (2020) [34]	Retrospective study	108	Unresectable HCC	TACE+MWA	TACE+RFA	Median survival of both the groups (20.9 months and 13 months) with unresectable HCC was increased. TACE combined with MWA also decreased the rate of complication occurrence (66% vs. 88.3%).
Yoon et al. (2018) [36]	Randomized clinical trial	90	Liver confined HCC showing macrovascular invasion	TACE +RT	Sorafenib	RT offered better progression-free survival (87% vs. 34.3%) than sorafenib and was very well tolerated with a higher radiological response rate (33.3% vs. 2.2%) and overall survival (55 weeks vs. 43 weeks).
Kalva et al. (2011) [39]	Retrospective study	54	Unresectable HCC	DEB-TACE	-	DEB-TACE was effective with better median survival at six months, one year, and two years at a percentage of 77%, 59%, and 32%, respectively, and overall median survival of 95%; DEB-TACE was well tolerated in patients with unresectable HCC.
Kudo et al. (2020) [41]	Randomized, multicentre, prospective trial	156	Unresectable HCC	TACE + sorafenib	TACE	Patients with TACE combined with sorafenib had a significant median progression-free survival (25.2 months vs. 13.5 months), the median time to untreatable progression (TTUP) ( 26.7 months vs. 20.6 months), and better overall survival at one and two years (96.2% and 82.7%) than TACE alone (77.2% and 64.6%).
Britten et al. (2012) [42]	Pilot study	30	HCC	TACE+ Bevacizumab	-	TACE when used in combination with bevacizumab, demonstrated less neovascularity (14% vs. 33%) compared to patients who had undergone TACE alone.
Finn et al. (2020) [43]	Interventional, global, open-label, phase III trial	336	Unresectable HCC without prior systemic therapy	Atezolizumab+ Bevacizumab	Sorafenib	Patients are given atezolizumab combined with bevacizumab, which resulted in increased median overall survival (6.8 months vs. 4.3 months) and progression-free survival at 12 months (67.2% vs. 56.6%) compared to sorafenib.

Bevacizumab, a monoclonal antibody targeting endothelial VEGF, was the first anti-angiogenic agent to be evaluated in patients with unresectable HCC with TACE [40]. Britten et al. conducted a pilot study with 30 patients with HCC, where one group was subjected to TACE alone and the other to TACE with bevacizumab; the study showed that TACE when used with bevacizumab, demonstrated less neovascularity (14% vs. 33%) when compared to patients who had undergone TACE alone (Table 5) [42]. Bevacizumab can also stabilize tumor vasculature and enhance tumor oxygenation, making them susceptible to chemotherapy and optimizing intra-tumoral pressure [40]. The United States Food and Drug Administration (FDA) in 2007 considered atezolizumab, a programmed death-ligand 1 (PD-L1) inhibitor in combination with bevacizumab to treat unresectable HCC. An interventional, global, open-label, phase three trial with a sample population of 336 patients with unresectable HCC in which one group received atezolizumab-bevacizumab and the other group received sorafenib. The study found that patients who were given atezolizumab combined with bevacizumab resulted in increased median overall survival (6.8 months vs. 4.3 months) and progression-free survival at 12 months (67.2% vs. 56.6%) compared to sorafenib (Table 5) [43]. In combination with immunotherapy, TACE is proven to be clinically beneficial [44]. Studies are currently being conducted on the usage of TACE with a combination of atezolizumab-bevacizumab in patients with HCC [45].

Role of TACE in neoadjuvant chemoembolization

TACE is also used in neoadjuvant chemoembolization pre-operatively to reduce the tumor's size and inhibit spread before HR or LT; adjuvant therapy aims to minimize the recurrence of HCC post-resection [12]. Zang et al. conducted a retrospective study for five years with a sample population of 1457 patients with HCC that underwent resection, including 120 patients treated pre-operatively with TACE. The study showed that compared to patients show had undergone resection alone, the patients who had pre-operative TACE demonstrated a five-year disease-free survival (Table 6) [46]. Another retrospective study conducted by Choi et al. for seven years on a sample population of 273 patients with HCC on the influence of pre-operative TACE showed that patients who had received TACE showed survival rates of one, three, and five years (76%, 57.7%, and 51.3%, respectively). In contrast, patients without TACE showed 20%, 53.8%, and 46.8% survival rates, respectively (Table 6) [47]. TACE is the most commonly used bridging therapy in LT patients while waiting for a suitable donor [48]. TACE decreased tumor growth and dissemination during the waiting period [49]. Graziadei et al. conducted a prospective study with a sample population of 41 patients with HCC who underwent TACE down staging before LT; the study showed that the survival rates of these patients post-LT at one, two, and five years were respectively 98%, 98% and 93% (Table 6) [50]. The summary of all the studies related to the utilization of TACE in neoadjuvant chemoembolization is summarized in Table 6.

**Table 6:** Role of TACE in neoadjuvant chemoembolization. HCC: hepatocellular carcinoma; LT: liver transplantation; TACE: transarterial chemoembolization

References	Design	Subjects	Criteria for inclusion	Pre-operative/post-operative TACE	Outcome
Zhang et al. (2000) [46]	Retrospective study	1725	Patients with HCC who are going to undergo hepatectomy	Pre-operative TACE	The patients who had pre-operative TACE demonstrated five-year disease-free survival.
Choi et al. (2007) [47]	Retrospective study	273	Patients with HCC who are going to undergo resection	Pre-operative TACE	Patients who had received TACE showed survival rates of one, three, and five years - 76%, 57.7%, and 51.3%, respectively; in contrast, patients without TACE showed 20%, 53.8%, and 46.8% survival rates, respectively.
Graziadei et al. (2003) [50]	Prospective study	41	Patients with HCC who are going to undergo LT	Pre-operative	Survival rates of the patients post-LT at one, two, and five years were 98%, 98%, and 93%, respectively.

Outcome

TACE is the most universally used therapy for unresectable HCC, including BCLC-C and Child-Pugh-B; it can improve survival and responsiveness without compromising hepatic functional reserve when done effectively (Table 1) [51,52]. According to EASL criteria for tumors, TACE induces a radiological complete response in 35% of the patients and 25% histological complete response in excised tumor or explanted liver [53,54]. TACE is also said to increase the five-year survival in patients with unresectable HCC [55]. Albumin >35 g/dl is the sole predictor for five-year survival, and a platelet count of 150 × 10^9^/l was associated with long-term survival in post-TACE patients [55]. Repeated TACE in patients with HCC was determined by patient tolerance to the current regimen; this technique has been more effective in lowering procedure-related morbidity and fatality rates [56]. The patients who underwent TACE had a fine line between the therapeutic impact of HCC and unwanted damage to the normal hepatic tissues [57]. The most prevalent adverse effects post-TACE was related to the post-embolization syndrome, which is characterized by fever, abdominal pain, and leucocytosis after the embolization of hepatic tumors; other complications are abnormal liver enzymes, vomiting, and nausea [58,59]. A brief course of steroids was recently proven to minimize the incidence of post-embolization syndrome [52]. High and widespread tumor burden is the main limiting factor as early diagnosis of HCC in a cirrhotic liver is difficult despite ongoing advances in imaging tools [60]. Another significant limiting factor is the high degree of variation in the cellular makeup of HCCs and the patient population [60]. Patients with HCC should be thoroughly evaluated prior to TACE to avoid tumor growth from interventional methods [61]. AASLD recommends that patients with functional liver decompensation be omitted as the hypoxic insult might worsen the viable hepatic tissues (Table 3) [7]. The absolute contraindication of using TACE is decompensated liver (Child-Pugh B) with tumor-related symptoms, impaired portal blood flow, portal vein thrombosis, extensive tumor, and creatinine >2 mg/dl [62]. TACE's relative contraindications are large tumors >10 cm, co-morbidities, biliary dilation, and untreated esophageal varices related to liver cirrhosis with a high risk of bleeding [62]. Even though TACE has been utilized for many decades, little progress has been made in comprehending the complicated local pharmacology, tumor heterogeneity within the population, and resistance to mechanism [44].

Disadvantages of TACE

TACE covers a wide range of therapeutic indications in hepato-oncology and when used correctly, it is a safe and effective therapy option [63]. Although TACE is generally a safe technique, it can have consequences; the most prevalent is acute cholecystitis [64]. Other problems associated with the procedure include hepatic abscess, pulmonary embolism, gastric mucosa injury, bile duct damage, and less commonly, severe pancreatitis [64]. The high risk of tumor recurrence is a significant drawback of all TACE regimens; intra-hepatic local recurrence in patients who developed early recurrence occurred more commonly in HCC patients who underwent TACE alone than those who had TACE combined with RFA [65]. In patients with early-stage HCC who had palliative TACE, heterogeneous lipiodol uptake, elevated serum DCP, and numerous tumors showed to be risk factors for recurrence [66]. One-year mortality is not rare in individuals with intermediate-stage HCC treated only with TACE. A high blood AFP level (> 400 ng/ml), Child-Pugh B cirrhosis, and tumor size are all independent risk factors for one-year death in such individuals [67]. Current treatment-related mortality associated with TACE is less than 1%, with acute liver insufficiency being the most common reason for death [55,58]. Oral administration of a nutritional supplement supplemented with branched-chain amino acids enhanced liver function following HCC resection and is being tested in patients undergoing TACE [68]. TACE should be discontinued in patients who develop an untreatable progression, such as developing contraindications to TACE once the regimen begins, deterioration of hepatic function or performance status post-TACE, or failure to elicit an objective response in the tumor after at least two treatments [69].

Limitations

This study does not address the efficacy of the wide variety of chemotherapy drugs used in combination with TACE; additionally, it does not address the usage of TACE in recurrent HCC.

## Conclusions

As evident in the studies mentioned in the review, despite the heterogeneity in HCC patients, TACE has shown to be beneficial in treating unresectable HCC. It has also led to being helpful in early-stage tumors where it is utilized as an alternative treatment when the criteria for the treatment of early-stage tumor according to the guidelines for the management are not met. TACE is also used to downstage before hepatectomy or as bridging therapy in patients before liver transplantation in advanced-stage tumors. TACE in combination therapy with chemotherapeutic drugs or anti-angiogenic agents was demonstrated to be very efficacious in inoperable HCC. However, prior to considering TACE as a treatment option in patients with unresectable HCC, the patient's risk profile, comorbidities, and treatment prognosis and benefit should be considered to increase the overall survival and decrease the occurrence of adverse events. As etiology and tumor burden fluctuates from patient to patient, managing patients with HCC remains challenging; thus, further studies are required that will undoubtedly widen the use of TACE and its use with different chemotherapeutic agents in patients with HCC.

## References

[1] Moradpour D, Blum HE (2005). Pathogenesis of hepatocellular carcinoma. Eur J Gastroenterol Hepatol.

[2] Grandhi MS, Kim AK, Ronnekleiv-Kelly SM, Kamel IR, Ghasebeh MA, Pawlik TM (2016). Hepatocellular carcinoma: from diagnosis to treatment. Surg Oncol.

[3] Sung H, Ferlay J, Siegel RL, Laversanne M, Soerjomataram I, Jemal A, Bray F (2021). Global Cancer Statistics 2020: GLOBOCAN estimates of incidence and mortality worldwide for 36 cancers in 185 countries. CA Cancer J Clin.

[4] Balogh J, Victor D 3rd, Asham EH (2016). Hepatocellular carcinoma: a review. J Hepatocell Carcinoma.

[5] Anthony PP (2001). Hepatocellular carcinoma: an overview. Histopathology.

[6] Di Bisceglie AM (2002). Epidemiology and clinical presentation of hepatocellular carcinoma. J Vasc Interv Radiol.

[7] Marrero JA, Kulik LM, Sirlin CB (2018). Diagnosis, staging, and management of hepatocellular carcinoma: 2018 practice guidance by the American Association for the Study of Liver Diseases. Hepatology.

[8] (2018). EASL clinical practice guidelines: management of hepatocellular carcinoma. J Hepatol.

[9] Osho A, Rich NE, Singal AG (2020). Role of imaging in management of hepatocellular carcinoma: surveillance, diagnosis, and treatment response. Hepatoma Res.

[10] Tzartzeva K, Obi J, Rich NE (2018). Surveillance imaging and alpha fetoprotein for early detection of hepatocellular carcinoma in patients with cirrhosis: a meta-analysis. Gastroenterology.

[11] Schwartz M, Roayaie S, Konstadoulakis M (2007). Strategies for the management of hepatocellular carcinoma. Nat Clin Pract Oncol.

[12] Chua TC, Liauw W, Saxena A, Chu F, Glenn D, Chai A, Morris DL (2010). Systematic review of neoadjuvant transarterial chemoembolization for resectable hepatocellular carcinoma. Liver Int.

[13] Rammohan A, Sathyanesan J, Ramaswami S (2012). Embolization of liver tumors: past, present and future. World J Radiol.

[14] Guan YS, He Q, Wang MQ (2012). Transcatheter arterial chemoembolization: history for more than 30 years. ISRN Gastroenterol.

[15] Park YN, Yang CP, Fernandez GJ, Cubukcu O, Thung SN, Theise ND (1998). Neoangiogenesis and sinusoidal "capillarization" in dysplastic nodules of the liver. Am J Surg Pathol.

[16] Murata S, Mine T, Ueda T, Nakazawa K, Onozawa S, Yasui D, Kumita S (2013). Transcatheter arterial chemoembolization based on hepatic hemodynamics for hepatocellular carcinoma. ScientificWorldJournal.

[17] Friedman MA, Volberding PA, Cassidy MJ, Resser KJ, Wasserman TH, Phillips TL (1979). Therapy for hepatocellular cancer with intrahepatic arterial adriamycin and 5-fluorouracil combined with whole-liver irradiation: a Northern California Oncology Group Study. Cancer Treat Rep.

[18] Misra NC, Jaiswal MS, Singh RV, Das B (1977). Intrahepatic arterial infusion of combination of mitomycin-c and 5-fluorouracil in treatment of primary and metastatic liver carcinoma. Cancer.

[19] Hirose H, Aoyama M, Oshima K (1982). Chemotherapy of hepatocellular carcinoma--with special reference to one-shot intra-arterial infusion of a high dose of adriamycin. [Article in Japanese]. Gan To Kagaku Ryoho.

[20] Chang Y, Jeong SW, Young Jang J, Jae Kim Y (2020). Recent updates of transarterial chemoembolilzation in hepatocellular carcinoma. Int J Mol Sci.

[21] Wáng YX, De Baere T, Idée JM, Ballet S (2015). Transcatheter embolization therapy in liver cancer: an update of clinical evidences. Chin J Cancer Res.

[22] Imai N, Ishigami M, Ishizu Y (2014). Transarterial chemoembolization for hepatocellular carcinoma: a review of techniques. World J Hepatol.

[23] Sreeramoju P, Libutti SK (2010). Strategies for targeting tumors and tumor vasculature for cancer therapy. Adv Genet.

[24] Reig M, Forner A, Rimola J (2022). BCLC strategy for prognosis prediction and treatment recommendation: the 2022 update. J Hepatol.

[25] Bruix J, Sherman M (2011). Management of hepatocellular carcinoma: an update. Hepatology.

[26] Han K, Kim JH (2015). Transarterial chemoembolization in hepatocellular carcinoma treatment: Barcelona clinic liver cancer staging system. World J Gastroenterol.

[27] Pons F, Varela M, Llovet JM (2005). Staging systems in hepatocellular carcinoma. HPB (Oxford).

[28] Oken MM, Creech RH, Tormey DC, Horton J, Davis TE, McFadden ET, Carbone PP (1982). Toxicity and response criteria of the Eastern Cooperative Oncology Group. Am J Clin Oncol.

[29] Hsu CY, Lee YH, Hsia CY (2013). Performance status in patients with hepatocellular carcinoma: determinants, prognostic impact, and ability to improve the Barcelona clinic liver cancer system. Hepatology.

[30] Pugh RN, Murray-Lyon IM, Dawson JL, Pietroni MC, Williams R (1973). Transection of the oesophagus for bleeding oesophageal varices. Br J Surg.

[31] Ren Y, Cao Y, Ma H (2019). Improved clinical outcome using transarterial chemoembolization combined with radiofrequency ablation for patients in Barcelona clinic liver cancer stage A or B hepatocellular carcinoma regardless of tumor size: results of a single-center retrospective case control study. BMC Cancer.

[32] Varghese J, Kedarisetty CK, Venkataraman J (2017). Combination of TACE and sorafenib improves outcomes in BCLC stages B/C of hepatocellular carcinoma: a single centre experience. Ann Hepatol.

[33] Xiang X, Lau WY, Wu ZY (2019). Transarterial chemoembolization versus best supportive care for patients with hepatocellular carcinoma with portal vein tumor thrombus: a multicenter study. Eur J Surg Oncol.

[34] Wei J, Cui W, Fan W, Wang Y, Li J (2020). Unresectable hepatocellular carcinoma: transcatheter arterial chemoembolization combined with microwave ablation vs. combined with cryoablation. Front Oncol.

[35] Kong JY, Li SM, Fan HY, Zhang L, Zhao HJ, Li SM (2018). Transarterial chemoembolization extends long-term survival in patients with unresectable hepatocellular carcinoma. Medicine (Baltimore).

[36] Yoon SM, Ryoo BY, Lee SJ (2018). Efficacy and safety of transarterial chemoembolization plus external beam radiotherapy vs sorafenib in hepatocellular carcinoma with macroscopic vascular invasion: a randomized clinical trial. JAMA Oncol.

[37] Liao M, Huang J, Zhang T, Wu H (2013). Transarterial chemoembolization in combination with local therapies for hepatocellular carcinoma: a meta-analysis. PLoS One.

[38] Taymaz-Nikerel H, Karabekmez ME, Eraslan S, Kırdar B (2018). Doxorubicin induces an extensive transcriptional and metabolic rewiring in yeast cells. Sci Rep.

[39] Kalva SP, Iqbal SI, Yeddula K, Blaszkowsky LS, Akbar A, Wicky S, Zhu AX (2011). Transarterial chemoembolization with doxorubicin-eluting microspheres for inoperable hepatocellular carcinoma. Gastrointest Cancer Res.

[40] Liapi E, Geschwind JF (2012). Combination of local transcatheter arterial chemoembolization and systemic anti-angiogenic therapy for unresectable hepatocellular carcinoma. Liver Cancer.

[41] Kudo M, Ueshima K, Ikeda M (2020). Randomised, multicentre prospective trial of transarterial chemoembolisation (TACE) plus sorafenib as compared with TACE alone in patients with hepatocellular carcinoma: TACTICS trial. Gut.

[42] Britten CD, Gomes AS, Wainberg ZA (2012). Transarterial chemoembolization plus or minus intravenous bevacizumab in the treatment of hepatocellular cancer: a pilot study. BMC Cancer.

[43] Finn RS, Qin S, Ikeda M (2020). Atezolizumab plus bevacizumab in unresectable hepatocellular carcinoma. N Engl J Med.

[44] Ebeling Barbier C, Heindryckx F, Lennernäs H (2021). Limitations and possibilities of transarterial chemotherapeutic treatment of hepatocellular carcinoma. Int J Mol Sci.

[45] Ben Khaled N, Seidensticker M, Ricke J (2022). Atezolizumab and bevacizumab with transarterial chemoembolization in hepatocellular carcinoma: the DEMAND trial protocol. Future Oncol.

[46] Zhang Z, Liu Q, He J, Yang J, Yang G, Wu M (2000). The effect of preoperative transcatheter hepatic arterial chemoembolization on disease-free survival after hepatectomy for hepatocellular carcinoma. Cancer.

[47] Choi GH, Kim DH, Kang CM, Kim KS, Choi JS, Lee WJ, Kim BR (2007). Is preoperative transarterial chemoembolization needed for a resectable hepatocellular carcinoma?. World J Surg.

[48] Coletta M, Nicolini D, Benedetti Cacciaguerra A, Mazzocato S, Rossi R, Vivarelli M (2017). Bridging patients with hepatocellular cancer waiting for liver transplant: all the patients are the same?. Transl Gastroenterol Hepatol.

[49] Vogl TJ, Naguib NN, Nour-Eldin NE (2009). Review on transarterial chemoembolization in hepatocellular carcinoma: palliative, combined, neoadjuvant, bridging, and symptomatic indications. Eur J Radiol.

[50] Graziadei IW, Sandmueller H, Waldenberger P (2003). Chemoembolization followed by liver transplantation for hepatocellular carcinoma impedes tumor progression while on the waiting list and leads to excellent outcome. Liver Transpl.

[51] Piscaglia F, Ogasawara S (2018). Patient selection for transarterial chemoembolization in hepatocellular carcinoma: importance of benefit/risk assessment. Liver Cancer.

[52] Ogasawara S, Chiba T, Ooka Y (2018). A randomized placebo-controlled trial of prophylactic dexamethasone for transcatheter arterial chemoembolization. Hepatology.

[53] Okayasu I, Hatakeyama S, Yoshida T, Yoshimatsu S, Tsuruta K, Miyamoto H, Kimula Y (1988). Selective and persistent deposition and gradual drainage of iodized oil, Lipiodol in the hepatocellular carcinoma after injection into the feeding hepatic artery. Am J Clin Pathol.

[54] Kudo M (2016). Regorafenib as second-line systemic therapy may change the treatment strategy and management paradigm for hepatocellular carcinoma. Liver Cancer.

[55] O'Suilleabhain CB, Poon RT, Yong JL, Ooi GC, Tso WK, Fan ST (2003). Factors predictive of 5-year survival after transarterial chemoembolization for inoperable hepatocellular carcinoma. Br J Surg.

[56] Ernst O, Sergent G, Mizrahi D, Delemazure O, Paris JC, L'Herminé C (1999). Treatment of hepatocellular carcinoma by transcatheter arterial chemoembolization: comparison of planned periodic chemoembolization and chemoembolization based on tumor response. AJR Am J Roentgenol.

[57] Millonig G, Graziadei IW, Freund MC (2007). Response to preoperative chemoembolization correlates with outcome after liver transplantation in patients with hepatocellular carcinoma. Liver Transpl.

[58] Romero Ubillus WJ, Munoz J, Vekaria M, Wollner IS, Getzen T (2011). Post-embolization syndrome: outcomes regarding the type of embolization. J Clin Oncol.

[59] Llovet JM, Bruix J (2003). Systematic review of randomized trials for unresectable hepatocellular carcinoma: chemoembolization improves survival. Hepatology.

[60] Galle PR, Tovoli F, Foerster F, Wörns MA, Cucchetti A, Bolondi L (2017). The treatment of intermediate stage tumours beyond TACE: from surgery to systemic therapy. J Hepatol.

[61] Jiang G, Ling S, Zhan Q, Zhuang L, Xu X (2021). Downstaging treatment for patients with hepatocelluar carcinoma before transplantation. Transplant Rev (Orlando).

[62] Sieghart W, Hucke F, Peck-Radosavljevic M (2015). Transarterial chemoembolization: modalities, indication, and patient selection. J Hepatol.

[63] Facciorusso A, Licinio R, Muscatiello N, Di Leo A, Barone M (2015). Transarterial chemoembolization: evidences from the literature and applications in hepatocellular carcinoma patients. World J Hepatol.

[64] Marcacuzco Quinto A, Nutu OA, San Román Manso R (2018). Complications of transarterial chemoembolization (TACE) in the treatment of liver tumors. Cir Esp (Engl Ed).

[65] Douhara A, Namisaki T, Moriya K (2017). Predisposing factors for hepatocellular carcinoma recurrence following initial remission after transcatheter arterial chemoembolization. Oncol Lett.

[66] Kinugasa H, Nouso K, Takeuchi Y (2012). Risk factors for recurrence after transarterial chemoembolization for early-stage hepatocellular carcinoma. J Gastroenterol.

[67] Lin CL, Hsieh CF, Chen T (2014). Risk factors for 1-year mortality in patients with intermediate-stage hepatocellular carcinoma treated solely with transcatheter arterial chemoembolization. Adv Dig Med.

[68] Chen L, Chen Y, Wang X (2015). Efficacy and safety of oral branched-chain amino acid supplementation in patients undergoing interventions for hepatocellular carcinoma: a meta-analysis. Nutr J.

[69] Lencioni R, Petruzzi P, Crocetti L (2013). Chemoembolization of hepatocellular carcinoma. Semin Intervent Radiol.

